# Improved Hypertension Control with the Imidazoline Agonist Moxonidine in a Multinational Metabolic Syndrome Population: Principal Results of the MERSY Study

**DOI:** 10.1155/2013/541689

**Published:** 2013-02-25

**Authors:** Irina Chazova, Markus P. Schlaich

**Affiliations:** ^1^Clinical Cardiology Institute A.L. Myasnikov FSI Russian Cardiology Scientific and Production Complex Ministry of Health, 3rd Cherepkovskaya No. 15, Moscow 121552, Russia; ^2^Neurovascular Hypertension and Kidney Disease Laboratory, Baker IDI Heart and Diabetes Institute, Melbourne, VIC 8008, Australia

## Abstract

This study was designed to assess the effects of moxonidine on blood pressure and aspects of the metabolic syndrome in racially diverse population of patients encountered in routine medical practice. Physicians collected data on a minimum of three consecutive patients with uncontrolled essential hypertension and criteria for metabolic syndrome, eligible to receive moxonidine (0.2–0.4 mg once daily) for 6 months, either as monotherapy or as adjunct therapy to current antihypertensive treatment. Systolic and diastolic blood pressure (BP) declined by an average of 24.5 + 14.3 mmHg and 12.6 + 9.1 mmHg, respectively. BP responder rates defined as attaining BP < 140/90 mmHg were significantly (*P* < 0.001) and substantially higher among younger patients, nonpostmenopausal women, and patients receiving monotherapy. While potentially relevant improvements in the entire cohort were observed in regard to body weight (−2.1 ± 5.4 kg), fasting plasma glucose (from 6.8 to 6.2 mmol/L), and triglycerides (2.4 to 2.0 mmol/L), statistically significant changes in metabolic parameters could only be detected in subgroup analyses. Moxonidine therapy reduced blood pressure and improved rates of blood pressure control in this group of patients. While the observed trend towards improvement in various metabolic parameters merits further investigation, the overall effect of moxonidine treatment is consistent with a reduction of total cardiovascular risk in this hypertensive metabolic syndrome cohort.

## 1. Introduction

Hypertension is a major contributor to cardiovascular disease (CVD) risk, but a patient's global CVD risk is determined by the interplay of multiple risk factors. In particular, the grouping of risk factors—elevated blood pressure, abdominal obesity, dyslipidaemia, and abnormalities of glucose and insulin metabolism, commonly referred to as metabolic syndrome has been associated with a substantial worsening of cardiovascular prognosis and all-cause mortality [1]. Postmenopausal women may be particularly susceptible to the development of metabolic syndrome and to its CVD consequences [2–5], including resistance to antihypertensive therapy [6].

Abdominal obesity is not only a cardinal feature of the metabolic syndrome but also an important contributor to the development and progression of cardiovascular and metabolic disturbances linked to the syndrome. Overactivity of the sympathetic nervous system (SNS) is of particular importance in this context. The interplay between obesity, elevated SNS activity, and hypertensive target organ damage is already demonstrable in very young overweight or obese adults [7, 8]. Elevated blood pressure may be initiated and sustained by increased SNS activation, as are metabolic alterations, inflammatory pathways, and target organ damage [8]. 

Targeting the SNS directly, therefore, provides a logical and attractive therapeutic target [7–12] in that it simultaneously addresses several relevant elements of the metabolic syndrome and therefore would be expected to reduce overall CVD risk to a greater extent than its isolated effect on blood pressure (BP) might predict. 

Moxonidine is a widely approved antihypertensive drug that lowers BP primarily by reducing central SNS activity via activation of imidazoline type-1 receptors in the rostral ventrolateral medulla [13]. In addition to its efficacy as an antihypertensive, moxonidine has been shown to improve indices of glycaemic control, aspects of the plasma lipid profile, and inflammatory markers [14–16]. Furthermore, its use has been associated with reduction in body weight [16–21]. The unique profile of this agent may provide an opportunity to simultaneously address a large number of factors crucially involved in the pathophysiology of the metabolic syndrome associated with elevated blood pressure and may offer several benefits in the management of hypertensive patients with metabolic syndrome. 

To this end, we conducted a large, multinational study to appraise the effects of moxonidine on blood pressure, anthropometric, lipid, and metabolic parameters of the metabolic syndrome in patients encountered in routine (real world) medical practice.

## 2. Methods 

### 2.1. Study Design

MERSY was a multinational, open-label, observational study with a planned duration of 6 months. Participating physicians were asked to collect data on a minimum of three consecutive patients with uncontrolled essential hypertension and metabolic syndrome, for whom moxonidine might be prescribed.

The primary objective was to evaluate the long-term safety and efficacy of moxonidine in hypertensive patients with metabolic syndrome. The secondary objective was to assess the effect of long-term treatment of moxonidine on laboratory parameters associated with the metabolic syndrome.

Moxonidine was prescribed at a dose of 0.2–0.4 mg once daily, either as monotherapy or as adjunctive therapy when the current antihypertensive treatment (which was to the discretion of the treating physician) was insufficient to achieve individual blood pressure targets or if it was not tolerated. The preferred maintenance dose of moxonidine was 0.4 mg/day, but physicians were permitted to initiate therapy at 0.2 mg and titrate to 0.4 mg/day after 2 weeks. After the baseline visit, a first follow-up visit was scheduled for between 1 and 3 months, according to the treating physician's usual practice for such consultations. A final visit was planned 6 months after starting moxonidine therapy. The study had no formal mechanisms to monitor compliance.

Adult patients (age ≥ 18 years) of either sex were eligible for enrolment if they had essential hypertension of any grade, as defined by the 2003 guidelines of the European Society of Hypertension. Patients were either newly diagnosed as hypertensive or had BP levels that were above target despite the use of other antihypertensive drugs measured according to the 2003 guidelines of the European Society of Hypertension or had failed to tolerate current antihypertensive treatment. For Australia only, supplementary inclusion criteria specified age not greater than 75 years and the persistence of hypertension despite concurrent antihypertensive therapy.

Criteria for a diagnosis of metabolic syndrome were based on the 2005 definition proposed by the International Diabetes Federation and comprised central obesity (defined as waist circumference ≥94 cm for Europid men and ≥80 cm for Europid women, with ethnicity-specific values for other groups) plus any two of the following: triglyceride (TG) levels ≥150 mg/dL (≥1.7 mmol/L) or specific treatment for this lipid abnormality; high-density lipoprotein (HDL) cholesterol <40 mg/dL (<1.03 mmol/L) in men or <50 mg/dL (<1.29 mmol/L) in women, or specific treatment for this lipid abnormality; systolic BP (SBP) ≥ 130 mmHg or diastolic BP (DBP) ≥ 85 mmHg, or treatment of previously diagnosed hypertension;fasting plasma glucose (FPG) ≥ 100 mg/dL (≥5.6 mmol/L) or previously diagnosed type 2 diabetes. If FPG was >5.6 mmol/L (>100 mg/dL), an oral glucose tolerance test was recommended, but this was not compulsory. Specific advice on lifestyle modification was not mandated by the protocol. 

The only criterion prohibiting patients from taking part in this study was the presence of contraindications to moxonidine, as identified in the relevant National Summary of Product Characteristics (SPC). 

### 2.2. Statistics and Data Analysis

From observations and experience in a previous postmarketing surveillance study [21], it was estimated that a study population of 2488 patients would be required for satisfactory statistical power. In order to allow for dropout and loss of data, a recruitment target of 3600 patients, recruited via 1200 physicians, was specified.

Nominal qualitative variables were compared using the *χ* ^2^ test or Fisher's exact test. Ordinal qualitative variables were compared using the Wilcoxon test or the Kruskal-Wallis test. Quantitative variables were compared using variance analysis.

BP, laboratory and weight parameters were compared between visits using covariance analysis, with the baseline value as the adjusted variable.

The absolute changes in heart rate (HR) between the baseline and postbaseline visits were summarized and analyzed through a one-sample *t*-test. All tests were two sided, with significance declared at the 5% level.

In addition to pooled results, subgroup analyses were undertaken according to menopause status (as determined by questioning), type of antihypertensive regimen (monotherapy or combination therapy), and age (<65 years and ≥65 years).

Data management and statistical analysis were conducted by the FOVEA Group, Rueil-Malmaison, France. Data entry was performed using Access version 9.0. Double data entry was used. Entered data were verified against case record form data when a discrepancy was found during double data entry. Quality control was performed using SAS version 8.2.

### 2.3. Efficacy Endpoints

The primary efficacy variable was the percentage of patients responding to antihypertensive therapy during the study. A response was defined as attainment of systemic arterial BP < 140/90 mmHg from baseline to each follow-up visit. BP limits of <130/80 mmHg were set for patients with a diagnosis of diabetes at baseline.

Secondary efficacy variables comprised the absolute change in BP from baseline to each follow-up visit, the absolute change in laboratory parameters for metabolic syndrome (FPG, TG, total cholesterol, HDL-cholesterol, low-density lipoprotein (LDL)-cholesterol, and creatinine, urinary albumin) from baseline to final visit at 6 months, and the absolute change in weight parameters (body mass index (BMI), waist-hip circumferences) from baseline to each follow-up visit.

### 2.4. Safety Endpoints

Suspect adverse drug reactions (SADRs)—defined as a response to a drug that was noxious and unintended and that occurred at doses normally used in humans for prophylaxis or treatment of a disease or to modify physical function—were screened for via active enquiry during follow-up and final visits. Each SADR was evaluated for duration, severity (mild, moderate, or severe), and seriousness. Physicians' assessment of the causal relationship to the investigational drug was documented, as was the action taken to address all SADRs and the outcome of each event. Special provisions were made for the reporting of all SADRs regarded as serious. Any pregnancies identified during the study were recorded and monitored as discrete events.

### 2.5. Administration and Ethical Considerations

The study was conducted in accordance with the ICH GCP (1997) and the Therapeutic Goods Administration (TGA) “Note for Guidance on Good Clinical Practice” (CPMP/ICH/135/95) annotated with TGA comments (July 2000). Patients were free to withdraw from the study at any time, for any reason, specified or unspecified, without prejudice to their medical care. Physicians were free to exclude any patient at any time if this was judged to be in the interests of the patient. 

## 3. Results

### 3.1. Patient Profile and Treatments

**Figure 1:**
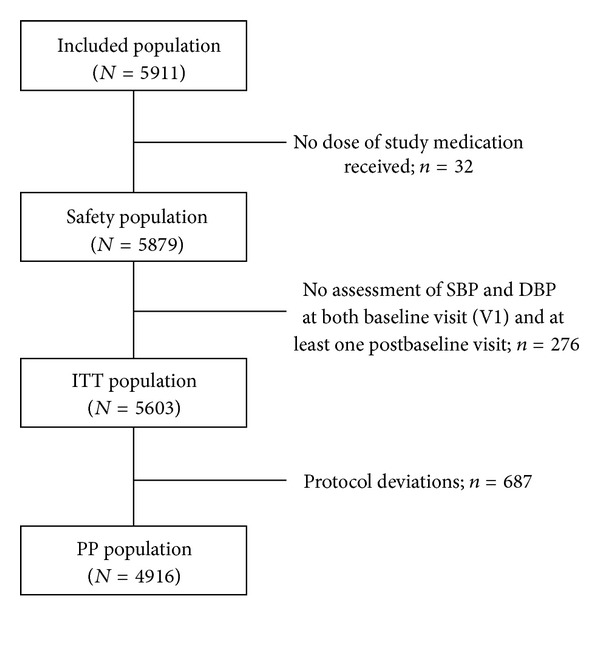
CONSORT summary of population recruitment.

The MERSY study was conducted in 13 countries between December 2006 and March 2008.  Figure 1 illustrates the derivation of the four patient populations. The present analysis reports primarily data from the intent-to-treat (ITT) (*N* = 5603) and safety (*n* = 5879) populations. Two countries (Bahrain and Switzerland) did not recruit any patients. 

Principal demographic details of the ITT population are depicted in Tables 1 and 2. Mean BP at baseline was (158.3 ± 13.8)/(94.1 ± 8.7)  mmHg. Mean SBP and DBP were ~4 mmHg lower in younger patients (<65 years) than in older ones ((157.6 ± 13.5)/(95.1 ± 8.4) mmHg versus (160.3 ± 14.4)/(91.6 ± 9.1) mmHg; *P* < 0.001), ~1 mmHg lower in nonpostmenopausal women (SBP 157.8 ± 13.7 mmHg versus 158.9 ± 14.1 mmHg; *P* < 0.001), and ~5 mmHg higher in patients receiving multiple antihypertensive medications than in those prescribed monotherapy ((159.2 ± 14.1)/(65.0 ± 13.4) mmHg versus (154.7 ± 11.7)/(60.9 ± 12.1) mmHg; *P* < 0.001 versus monotherapy). Differing national legislations precluded a complete audit of ethnicity data.

There was statistical evidence of variations in various metabolic syndrome-related metabolic parameters according to age, therapeutic regimen, and menopause status, but with the exception of FPG according to menopause status (6.9 ± 2.1 mmol/L in postmenopausal women versus 6.6 ± 2.1 mmol/L in nonpostmenopausal women) (*P* = 0.001), these differences were numerically small (data not shown).

In the month preceding the baseline visit, most patients (*n* = 3506) had received multiagent combination therapy for BP control. Diuretics were the single most widely prescribed class of drugs recorded in this subset of patients (*n* = 2277). A further 1200 patients had received monotherapy, of which 605 had received either an angiotensin-converting enzyme (ACE) inhibitor or an angiotensin receptor blocker (ARB), 199 had received a calcium-channel blocker, 160 had been prescribed a beta-blocker, and 142 had received a diuretic. A further 719 patients had received no antihypertensive medication during that month.

A baseline diagnosis of diabetes was present for 47.1% (2623) of the ITT population for whom data were available (*n* = 5567), with diabetes proportionately more often recorded in older patients (54.2% at age ≥ 65 years versus 44.8% at <65 years), those taking multiple antihypertensive drugs (51.5% versus 28.7% versus monotherapy), and postmenopausal women (50.5% versus 35.2% versus nonpostmenopausal) (*P* < 0.001 for all comparisons).

Documented reasons for initiating moxonidine therapy comprised lack of efficacy of current antihypertensive medication (*n* = 3885), intolerance to current antihypertensive medication (*n* = 286), or a new diagnosis of hypertension (*n* = 886); 138 cases were classified as “other reasons.” Lack of efficacy of current therapies was more likely to be the reason in older patients, postmenopausal patients, and patients taking multiple antihypertensive drugs, whereas a new diagnosis of hypertension was proportionately more common in younger nonpostmenopausal patients.

Moxonidine 0.2 mg/day was prescribed to 1731 patients at the baseline visit. Doses up to 0.4 mg/day were prescribed to a further 3635 patients. Among the 4118 patients of the ITT cohort for whom medication data were available from the last study visit, 20.0% (*n* = 823) were being prescribed moxonidine 0.2 mg/day at that time and 76.3% were prescribed doses up to 0.4 mg/day (*n* = 3143). The percentage of patients who received moxonidine as monotherapy (19-20%) or as part of multiple combination therapy (80-81%) remained constant throughout the study.

### 3.2. Primary Efficacy Endpoint

The proportion of patients classified as responding to hypertension therapy increased progressively during the study, from 24.2% (*n* = 1345) at first in-study clinical visit (between 1 and 3 months) to 41.3% (*n* = 2314) at final assessment at 6 months. (This total comprised nondiabetic patients achieving SBP < 140 mmHg and diabetes patients achieving SBP < 130 mmHg.) Responder rates were significantly (*P* < 0.001) and substantially higher among younger patients (44.3% versus 33.4% in older patients), nonpostmenopausal women (52.8% versus 38.5% in postmenopausal women), and patients receiving monotherapy (55.7% versus 37.8% in those receiving multidrug therapy).

**Figure 2:**
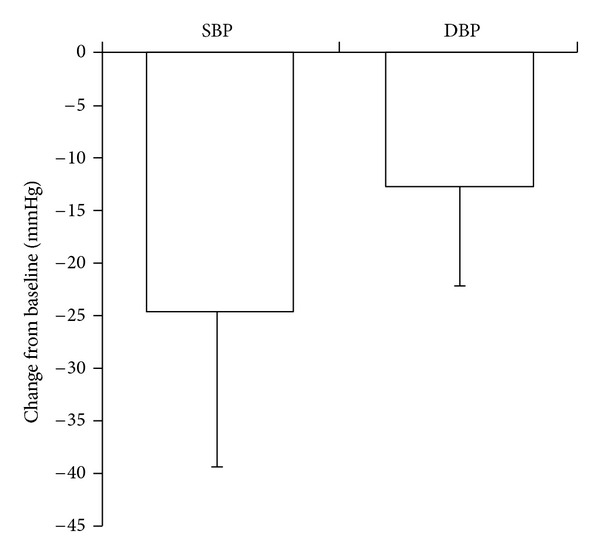
SBP and DBP responses during moxonidine therapy. Mean ± SD.

**Figure 3:**
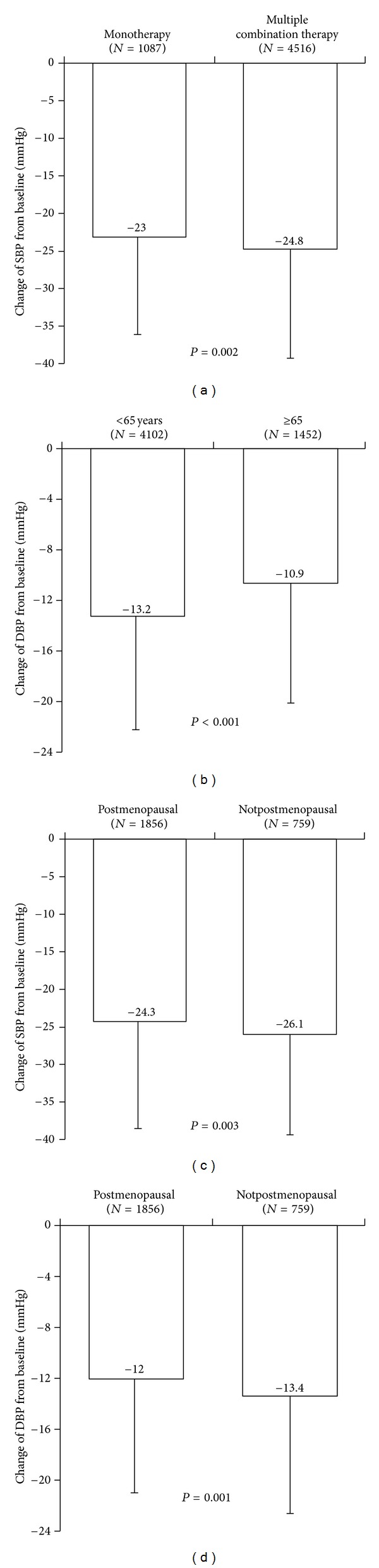
(a) Evolution of SBP by type of antihypertensive treatment (ITT), (b) evolution of DBP by age cohort between V1 and V3 (ITT population, *N* = 5603), (c) evolution of SBP by menopausal status (ITT population; *n* = 2772), and (d) evolution of DBP by menopausal status (ITT population; *n* = 2772).

SBP and DBP declined by an average of 24.5 ± 14.3 mmHg and 12.6 ± 9.1 mmHg, respectively, across the study, as illustrated in Figure 2. The mean change in pulse pressure was –11.8 ± 12.8 mmHg. As illustrated in Figures 3(a)–3(d), a range of mostly moderate but statistically significant variations in blood pressure changes were recorded in patient subgroups.

### 3.3. Secondary Efficacy Endpoints

On-treatment changes were recorded for mean values of every nominated laboratory parameter except creatinine (Table 3). The proportional changes in FPG did not differ significantly between prespecified subgroups (*P* > 0.2), notwithstanding differences in absolute values. There were likewise no subgroup-specific variations in the trend for total cholesterol, creatinine, or urinary albumin. 

By contrast, the reduction in TG and the increase in HDL-C levels was more marked in younger (versus older) patients, and the reduction in TGs was significantly larger in nonpostmenopausal women (versus postmenopausal) (*P* < 0.001) for all comparisons, except HDL-C (*P* = 0.004). The reduction in LDL-C during treatment was more marked in younger patients than older ones (*P* = 0.007).

Average weight declined by –2.1 ± 5.4 kg during the study and BMI declined by –0.7 ± 2.0 kg/m^2^. Mean HR, assessed in the safety population (*n* = 5879), fell from 79.6 ± 9.1 beats/min to 74.1 ± 7.0 beats/min, an average reduction of –5.7 ± 8.2.

Patients' assessments of treatment were “excellent,” “good,” “tolerable,” or “bad" in 44.4%, 48.3%, 6.4%, and 0.9% (*n* = 46) of cases, respectively. The distribution of investigators' impressions of treatment was similar.

### 3.4. Safety Findings

During the course of the study, 195 SADRs were recorded in 132 patients (2.2% of the study population) (Table 4). Of these events, 12 (in 6 patients) were classified as serious. Just under half led to study termination; the number of severe SADRs was small (*n* = 15). Events contributing at least 5% of the total SADR count comprised gastrointestinal disorders (55 events; 28.2%); nervous system disorders (53 events; 27.2%); general disorders and administration site conditions (29 events; 14.9%); and skin and subcutaneous tissue disorders (10 events; 5.1%). The most frequent SADRs linked with the gastrointestinal system were dry mouth (48 events in 47 subjects) while most of the SADRs linked with the nervous system included dizziness (16 events in 16 subjects) or headache (13 events in 13 subjects). Nervous system disorders were the single largest category of events associated with study termination and the single largest source of events rated as severe (*n* = 9).

Of the 12 serious SADRs, 2 each were classified as nervous system disorders; vascular disorders; infections and infestations; or respiratory, thoracic, and mediastinal disorders. The remaining four serious SADRs comprised one case each in the categories of psychiatric disorder; pregnancy, puerperium, and perinatal conditions; renal and urinary disorders; and cardiac disorders. 

No deaths were reported during the study.

## 4. Discussion 

This open-label phase IV trial was designed to assess the effect of moxonidine on BP and laboratory parameters associated with the metabolic syndrome after 6 months of treatment in a general practice setting. We sought to enlarge on previous experience in a single country [21] by recruiting our patients from 11 countries with varying ethnic and racial profiles. 

The results of the MERSY study are consistent with the previous experience with moxonidine in the management of hypertension [21–24]. The  ~16% increment in responder rates seen in our patient sample was smaller than was reported in a placebo-controlled assessment [25]. Such a difference might have been predicted given the more complex clinical circumstances of our patients, the high degree of treatment resistance at baseline, and the extensive use of moxonidine initially at doses <0.4 mg. Nevertheless, the earlier study [25] provides a useful placebo-controlled benchmark for assessing the scale of the response seen in our patients and persuades us that the improvement in responder rates was a true effect plausibly attributable to the use of moxonidine. We regard the close similarity in the absolute magnitude of SBP and DBP reductions in our patients and in the CAMUS study [21] as also noteworthy in this context. A blood pressure reduction of this magnitude compares favorably with that achieved by other antihypertensive drug classes considered as first choice such as diuretics and others, particularly as add-on treatment at doses commonly used. Recent reports demonstrating the failure to achieve blood pressure goals despite the widespread use of antihypertensive treatment in a multinational European survey of patients with metabolic syndrome highlights the need for more assertive and effective treatment of blood pressure in this growing segment of the adult population [26]. Data from the MERSY trial support the view that targeting the SNS centrally could be seen as part of the response to this need. 

While blood pressure reduction is the primary goal of antihypertensive therapy, potential effects on metabolic parameters also need to be taken into account. Such considerations have led to widespread recommendations in national and international guidelines to avoid beta-blockers and diuretics in patients with metabolic disturbances or diabetes mellitus if not indicated for additional comorbidities. The reasoning for these recommendations relates to the well-described weight gain with beta-blockers and the adverse metabolic effects (such as insulin resistance and hyperuricemia) encountered with both beta-blockers and diuretics. In contrast, antihypertensive agents that exert no or even beneficial metabolic effects, such as calcium channel blockers (considered neutral in this regard) and inhibitors of the renin-angiotensin-system (ACE inhibitors, angiotensin receptor blockers, and direct renin inhibitors), which have been shown to reduce new onset of diabetes [27], are considered preferred choices in this scenario. 

Moxonidine clearly falls into the second category, with proven efficacy in regard to blood pressure lowering and beneficial effects in regard to diverse metabolic parameters. While the effects of moxonidine on individual metabolic indices in our study could be considered as modest, the trends of all the changes seen were towards a profile of lower overall CVD risk, which was particularly evident in the subgroup analyses.

In this context, the average reduction in body weight of 2.1 kg in our patients is noteworthy and replicates earlier studies of moxonidine in populations with metabolic syndrome [16, 21]. Weight loss has clearly been associated with improved CV and other outcomes, suggesting that moxonidine may have additional beneficial effects beyond blood pressure reduction, particularly in overweight or obese hypertensive subjects or those with the metabolic syndrome. The recent withdrawal of sibutramine [28] and other primary weight-loss drugs emphasizes the desirability of having an agent with such an effect. 

Several lines of evidence suggest that sympathetic activation is of particular relevance in the earlier stages of hypertension [7, 8, 11]. Our data may provide additional support for these observations in that the BP responder rates were significantly (*P* < 0.001) and substantially higher among younger compared to older patients (44.3% versus 33.4%). Interestingly, the reduction in LDL-C and TG (*P* < 0.001) and the increase in HDL-C levels (*P* = 0.004) was also more marked in younger than in older patients. These findings may indicate that younger subjects derive specific benefit from centrally sympatholytic agents.

Our observation of reductions in TG and body weight in conjunction with improvement in blood pressure control are compatible with the suggestion [29] that visceral obesity and dyslipidaemia are central contributors to the resistance of hypertension in metabolic syndrome, but are not an a priori proof of this idea. Indeed, given that we observed advantageous trends in most of the metabolic indices measured, the precise elements of metabolic syndrome involved in blood pressure resistance may be immaterial if inhibition of central sympathetic outflow becomes part of the antihypertensive strategy. It, therefore, appears plausible to suggest that centrally acting sympatholytic agents such as moxonidine may be considered an equally effective and beneficial choice as inhibitors of the renin-angiotensin system in patients with hypertension and metabolic syndrome, perhaps a preferred choice when compared to calcium-channel blockers, and most likely preferable to beta-blockers and diuretics, if no other conditions warrant their use. The validity of this concept has recently been demonstrated by the results of a renal denervation technique to inhibit sympathetic nervous system activation in obese patients with resistant hypertension. Despite being applied on a background of multiple antihypertensive medications, this method achieved an average blood pressure reduction of 32/12 mmHg and was associated with significant improvements in glycemic control and insulin sensitivity [30].

Additional, albeit indirect evidence for this concept comes from the ALMAZ study [15], in which the beneficial effects of moxonidine on indices of glucose homoeostasis were most marked in patients with a heart rate >80 beats/min, considered as an indicator of increased sympathetic drive. We did not stratify responses to therapy by heart rate in MERSY but if heart rate is a valid proxy for SNS status, a 5 beats/min decline in heart rate in our cohort may have been relevant to the overall effects of moxonidine in our study. Since high heart rate may contribute to cardiovascular risk [31, 32], the demonstrated ability of moxonidine to lower heart rate may well be relevant to its therapeutic profile. Given the increasing number of studies indicating potential beneficial effects of moxonidine beyond those on BP and expanding to metabolic parameters, additional studies and meta-analyses of existing studies may be useful to confirm the validity of this concept. 

Moxonidine was well tolerated when used in combinations with the range of first-line antihypertensives in MERSY, as was the case in other studies. More generally, the safety profile of moxonidine in the MERSY study was fully in accordance with the known effects of the drug. The overall incidence of SADRs was very low (<2.5%) and the nature of the SADRs observed was consistent with previous experience. No previously unreported terms of SADR were encountered during our study. The incidence of SADRs with moxonidine is usually highest during the first weeks of treatment and thereafter declines to very low levels. The independent decision of many investigators to start therapy at doses <0.4 mg/day may have contributed to the excellent tolerability profile of moxonidine in the MERSY study and may be regarded as an example of skill in clinical practice.

## 5. Conclusions

In summary, in this large sample of patients with hypertension and concomitant metabolic syndrome, moxonidine enhanced blood pressure control when used alone or in combination and was associated with improvement in several aspects of metabolic syndrome. Moxonidine was well-tolerated during 6 months of continuous use. Given current estimations suggesting that approximately 70% of all incident hypertension is associated with overweight or obesity, it appears justified to recommend antihypertensive treatment with an agent that targets the underlying pathophysiology including sympathetic activation, particularly so if additional benefits can be achieved in regard to control of body weight and other metabolic markers characterizing the metabolic syndrome.

## Figures and Tables

**Table 1:** Summary demographic details of the intent-to-treat (ITT) population.

Total patients	*N* = 5603
Sex (*n* = 5554)	
Male	2793 (50.2%)
Female	2772 (49.8%)
Age (yrs) (*n* = 5554)	
<40	397 (7.1%)
40–49	1045 (18.8%)
50–59	1854 (33.4%)
60–69	1458 (26.2%)
>69	804 (14.5%)
<65	4102 (73.9%)
≥65	1452 (26.1%)
Menopause status (*n* = 2615)	
Postmenopausal	1856 (71.0%)
Non postmenopausal	759 (29.0%)
Height (mean ± SD, cm) (*n* = 5464)	168.1 ± 8.9
Weight (mean ± SD, kg) (*n* = 5464)	91.9 ± 15.6
BMI (mean ± SD, kg/m^2^) (*n* = 5464)	32.5 ± 5.0
Waist circumference (mean ± SD, cm) (*n* = 5195)	104.6 ± 13.3
Hip circumference (mean ± SD, cm) (*n* = 4722)	107.7 ± 13.7
Race/ethnicity (*n* = 4815)	
White	2312 (48.0%)
American Indian or Alaska native	1496 (31.1%)
Asian	835 (17.3%)
Black of African heritage or African American	149 (3.1%)
Native Hawaiian or other Pacific Islander	17 (0.4%)
Aboriginal/Torres Strait Islander	6 (0.1%)
Smoker status (*n* = 5453)	
Yes	1292 (23.7%)
No	4161 (76.3%)

**Table 2:** Baseline metabolic indices in the intent-to-treat (ITT) population. The sample sizes for variables are less than the full ITT population (*n* = 5603), due to lack of data*. *

	Means ± SD (mmol/L)
Fasting plasma glucose (*n* = 2551)	6.8 ± 2.1
Triglycerides (*n* = 2288)	2.4 ± 1.1
Cholesterol (*n* = 2305)	5.8 ± 1.1
HDL-cholesterol (*n* = 1893)	1.2 ± 0.5
LDL-cholesterol (*n* = 1421)	3.5 ± 1.1
Creatinine (*n* = 1952)	0.09 ± 0.06
Urinary albumin (*n* = 272)	92.7 ± 191.6

**Table 3:** In-study trends in laboratory parameters associated with the metabolic syndrome (secondary efficacy endpoints). All results expressed as mmol/L unless indicated otherwise. Data are expressed as mean ± SD.

Total patients	*N* = 5603
Fasting plasma glucose	
At study start	6.8 ± 2.1
At study end	6.2 ± 1.6
In-study change	−0.8 ± 1.6
Triglycerides	
At study start	2.4 ± 1.1
At study end	2.0 ± 0.9
In-study change	−0.6 ± 1.0
Cholesterol	
At study start	5.8 ± 1.1
At study end	5.2 ± 0.9
In-study change	−0.7 ± 1.0
HDL-cholesterol	
At study start	1.2 ± 0.5
At study end	1.3 ± 0.5
In-study change	0.1 ± 0.5
LDL-cholesterol	
At study start	3.5 ± 1.1
At study end	3.0 ± 0.9
In-study change	−0.5 ± 0.9
Creatinine	
At study start	0.09 ± 0.06
At study end	0.10 ± 0.07
In-study change	0.01 ± 0.04
Urinary albumin	
At study start	92.7 ± 191.6
At study end	83.3 ± 205.5
In-study change	−7.6 ± 153.1
Body weight (kg)	
At study start	92.0 ± 15.6
At study end	90.0 ± 15.3
In-study change	−2.1 ± 5.4

**Table 4:** Summary of suspected adverse drug reactions (SADRs) recorded during the study.

	No. of events	No. of patients (%)
All SADRs	195	132 (2.2%)
SADRs considered related to study treatment	151	97 (1.6%)
SADRs leading to study termination	93	62 (1.1%)
Severe SADRs	15	10 (0.2%)
Serious SADRs	12	6 (0.1%)

## References

[1] Mottillo S, Filion KB, Genest J (2010). The metabolic syndrome and cardiovascular risk a systematic review and meta-analysis. *Journal of the American College of Cardiology*.

[2] Lin JW, Caffrey JL, Chang MH, Lin YS (2010). Sex, menopause, metabolic syndrome, and all-cause and cause-specific mortality—cohort analysis from the third national health and nutrition examination survey. *Journal of Clinical Endocrinology and Metabolism*.

[3] Janssen I, Powell LH, Crawford S, Lasley B, Sutton-Tyrrell K (2008). Menopause and the metabolic syndrome: the study of women’s health across the nation. *Archives of Internal Medicine*.

[4] Phillips GB, Jing T, Heymsfield SB (2008). Does insulin resistance, visceral adiposity, or a sex hormone alteration underlie the metabolic syndrome? Studies in women. *Metabolism*.

[5] Regitz-Zagrosek V, Lehmkuhl E, Weickert MO (2006). Gender differences in the metabolic syndrome and their role for cardiovascular disease. *Clinical Research in Cardiology*.

[6] Rossi R, Nuzzo A, Origliani G, Modena MG (2008). Metabolic syndrome affects cardiovascular risk profile and response to treatment in hypertensive postmenopausal women. *Hypertension*.

[7] Lambert E, Sari CI, Dawood T (2010). Sympathetic nervous system activity is associated with obesity-induced subclinical organ damage in young adults. *Hypertension*.

[8] Lambert GW, Straznicky NE, Lambert EA, Dixon JB, Schlaich MP (2010). Sympathetic nervous activation in obesity and the metabolic syndrome—causes, consequences and therapeutic implications. *Pharmacology and Therapeutics*.

[9] Licht CMM, Vreeburg SA, Van Reedt Dortland AKB (2010). Increased sympathetic and decreased parasympathetic activity rather than changes in hypothalamic-pituitary-adrenal axis activity is associated with metabolic abnormalities. *Journal of Clinical Endocrinology and Metabolism*.

[10] Szczepanska-Sadowska E, Cudnoch-Jedrzejewska A, Ufnal M, Zera T (2010). Brain and cardiovascular diseases: common neurogenic background of cardiovascular, metabolic and inflammatory diseases. *Journal of Physiology and Pharmacology*.

[11] Mancia G, Bousquet P, Elghozi JL (2007). The sympathetic nervous system and the metabolic syndrome. *Journal of Hypertension*.

[12] Masuo K, Rakugi H, Ogihara T, Esler MD, Lambert GW (2010). Cardiovascular and renal complications of type 2 diabetes in obesity: role of sympathetic nerve activity and insulin resistance. *Current Diabetes Reviews*.

[13] Van Zwieten PA (1997). Centrally acting antihypertensives: a renaissance of interest. Mechanisms and haemodynamics. *Journal of Hypertension*.

[14] Pöyhönen-Alho MK, Manhem K, Katzman P (2008). Central sympatholytic therapy has anti-inflammatory properties in hypertensive postmenopausal women. *Journal of Hypertension*.

[15] Topal E, Cikim AS, Cikim K, Temel I, Ozdemir R (2006). The effect of moxonidine on endothelial dysfunction in metabolic syndrome. *American Journal of Cardiovascular Drugs*.

[16] Chazova I, Almazov VA, Shlyakhto E (2006). Moxonidine improves glycaemic control in mildly hypertensive, overweight patients: a comparison with metformin. *Diabetes, Obesity and Metabolism*.

[17] Ebinç H, Ozkurt ZN, Ebinç FA, Ucardag D, Caglayan O, Yilmaz M (2008). Effects of sympatholytic therapy with moxonidine on serum adiponectin levels in hypertensive women. *Journal of International Medical Research*.

[18] Kaaja R, Kujala S, Manhem K (2007). Effects of sympatholytic therapy on insulin sensitivity indices in hypertensive postmenopausal women. *International Journal of Clinical Pharmacology and Therapeutics*.

[19] Sanjuliani AF, De Abreu VG, Francischetti EA (2006). Selective imidazoline agonist moxonidine in obese hypertensive patients. *International Journal of Clinical Practice*.

[20] Derosa G, Cicero AFG, D’Angelo A (2007). Metabolic and antihypertensive effects of moxonidine and moxonidine plus irbesartan in patients with type 2 diabetes mellitus and mild hypertension: a sequential, randomized, double-blind clinical trial. *Clinical Therapeutics*.

[21] Sharma AM, Wagner T, Marsalek P (2004). Moxonidine in the treatment of overweight and obese patients with the metabolic syndrome: a postmarketing surveillance study. *Journal of Human Hypertension*.

[22] Prichard BNC, Simmons R, Rooks MJ, Haworth DA, Laws D, Wonnacott S (1992). A double-blind comparison of moxonidine and atenolol in the management of patients with mild-to-moderate hypertension. *Journal of Cardiovascular Pharmacology*.

[23] Wolf R (1992). the treatment of hypertensive patients with a calcium antagonist or moxonidine: a comparison. *Journal of Cardiovascular Pharmacology*.

[24] Trieb G, Jäger B, Hughes PR, Gardosch von Krosigk P (1995). Long-term evaluation of the antihypertensive efficacy and tolerability of the orally-acting imidazoline I_1_ receptor agonist moxonidine. *European Journal of Clinical Research*.

[25] Frei M, Kuster L, Von Krosigk PPG, Koch HF, Kuppers H (1994). Moxonidine and hydrochlorothiazide in combination: a synergistic antihypertensive effect. *Journal of Cardiovascular Pharmacology*.

[26] Kjeldsen SE, Naditch-Brule L, Perlini S, Zidek W, Farsang C (2008). Increased prevalence of metabolic syndrome in uncontrolled hypertension across Europe: the Global Cardiometabolic Risk Profile in Patients with hypertension disease survey. *Journal of Hypertension*.

[27] Schmieder RE, Hilgers KF, Schlaich MP, Schmidt BM (2007). Renin-angiotensin system and cardiovascular risk. *Lancet*.

[28] James WPT, Caterson ID, Coutinho W (2010). Effect of sibutramine on cardiovascular outcomes in overweight and obese subjects. *New England Journal of Medicine*.

[29] Zidek W, Naditch-Brûlé L, Perlini S, Farsang C, Kjeldsen SE (2009). Blood pressure control and components of the metabolic syndrome: the GOOD survey. *Cardiovascular Diabetology*.

[30] Mahfoud F, Schlaich M, Kindermann I (2011). Effect of renal sympathetic denervation on glucose metabolism in patients with resistant hypertension: a pilot study. *Circulation*.

[31] Palatini P (2009). Elevated heart rate in cardiovascular diseases: a target for treatment?. *Progress in Cardiovascular Diseases*.

[32] Fox K, Borer JS, Camm AJ (2007). Resting heart rate in cardiovascular disease. *Journal of the American College of Cardiology*.

